# Exploring the Association between Cathepsin B and Parkinson’s Disease

**DOI:** 10.3390/brainsci14050482

**Published:** 2024-05-10

**Authors:** Changhao Lu, Xinyi Cai, Shilin Zhi, Xiaofen Wen, Jiaxin Shen, Tommaso Ercoli, Elena Rita Simula, Carla Masala, Leonardo A. Sechi, Paolo Solla

**Affiliations:** 1 Department of Medical, Surgical and Experimental Sciences, University of Sassari, 07100 Sassari, Italy; c.lu@studenti.uniss.it; 2 Department of Biomedical Sciences, University of Sassari, 07100 Sassari, Italy; simulaelena@gmail.com (E.R.S.); sechila@uniss.it (L.A.S.); 3 Provincial Key Laboratory of Infectious Diseases and Molecular Immunopathology, Department of Pathology, Shantou University Medical College, Shantou 515041, China; 16xycai@stu.edu.cn; 4 Department of Gastrointestinal Surgery, Sun Yat-sen Memorial Hospital, Sun Yat-sen University, Guangzhou 510120, China; zhishl@mail2.sysu.edu.cn; 5 Department of Medical Oncology, Cancer Hospital of Shantou University Medical College, Shantou 515041, China; x.wen@studenti.uniss.it; 6 Department of Hematology, The First Affiliated Hospital of Shantou University Medical College, Shantou 515041, China; j.shen@studenti.uniss.it; 7 Department of Neurology, University of Sassari, Viale S. Pietro 10, 07100 Sassari, Italy; 8 Department of Biomedical Sciences, University of Cagliari, SP 8 Cittadella Universitaria, 09042 Monserrato, Italy; cmasala@unica.it; 9 Struttura Complessa di Microbiologia e Virologia, Azienda Ospedaliera Universitaria di Sassari, 07100 Sassari, Italy

**Keywords:** Parkinson’s Disease, Cathepsin B, *N*-acetylaspartate, mendelian randomization, mediating factor

## Abstract

Objective: The aim of this study is to investigate the association between Cathepsin B and Parkinson’s Disease (PD), with a particular focus on determining the role of *N*-acetylaspartate as a potential mediator. Methods: We used summary-level data from Genome-Wide Association Studies (GWAS) for a two-sample Mendelian randomization (MR) analysis, exploring the association between Cathepsin B (3301 cases) and PD (4681 cases). A sequential two-step MR approach was applied (8148 cases) to study the role of *N*-acetylaspartate. Results: The MR analysis yielded that genetically predicted elevated Cathepsin B levels correlated with a reduced risk of developing PD (*p* = 0.0133, OR: 0.9171, 95% CI: 0.8563–0.9821). On the other hand, the analysis provided insufficient evidence to determine that PD affected Cathepsin B levels (*p* = 0.8567, OR: 1.0035, 95% CI: 0.9666–1.0418). The estimated effect of *N*-acetylaspartate in this process was 7.52% (95% CI = −3.65% to 18.69%). Conclusions: This study suggested that elevated Cathepsin B levels decreased the risk of developing PD, with the mediation effect of *N*-acetylaspartate. Further research is needed to better understand this relationship.

## 1. Introduction

Parkinson’s Disease (PD) is the second most frequent neurodegenerative disorder, usually affecting individuals over 60 years, with a male prevalence [1,2,3]. As the disease progresses, PD patients increase the burden of their disability, a condition that cannot be stopped by the current treatment options [4]. Neuropathological findings have revealed that PD is mainly characterized by dopaminergic neuron loss in the substantia nigra of the midbrain [5].

PD is clinically characterized by cardinal motor symptoms such as bradykinesia, rigidity, tremor, and postural instability [6]. Moreover, it is well known that PD is also associated with non-motor symptoms (NMS), including olfactory disorders [7], apathy, fatigue, and cognitive impairment [8]. The diagnosis of PD relies on neurological examination since imaging techniques offer a limited diagnostic value [9,10]. The current management of PD patients is based on symptomatic actions since no disease-modifying treatment is currently available [11].

Recent studies emphasized a possible role of lysosomes in the etiopathogenesis of PD [12]. Indeed, lysosomes have been implicated in the cellular pathway that could regulate α-synuclein proteins, which are involved in the development of PD [13]. Within this context, Cathepsin B, a lysosomal hydrolase, has been indicated as a potential biomarker and risk gene for PD [14]. Studies across several different cohorts have reinforced the association between Cathepsin B and PD. For instance, Chang and colleagues performed a Genome-Wide Association Study (GWAS) of 6476 subjects from the 23 and Me PD cohort (PDWBS (Web-Based Study of Parkinson’s Disease)) and 302,042 controls genotyped on custom Illumina arrays [15], identifying that common variations in the CTSB gene, which encodes Cathepsin B, have been linked to an elevated PD risk. Moreover, Milanowski and collaborators performed a Whole Exome Sequencing (WES) analysis in a PD family, confirming the possible role of the CTSB gene [16]. Jones-Tabah and colleagues used genetic data from the Fox Investigation for New Discovery of Biomarkers (BioFIND), the Harvard Biomarker Study (HBS), the Parkinson’s Progression Markers Initiative (PPMI), the Parkinson’s Disease Biomarkers Program (PDBP), the International LBD Genomics Consortium (ILBDGC), and STEADY-PD III Investigators, observing that rare variations in the CTSB gene have been associated with an elevated risk of PD [17]. Despite the growing number of evidence, the association between circulating Cathepsin B levels and Parkinson’s Disease risk is still uncertain, primarily due to the small sample sizes of the studies, limited follow-up duration, and the potential risk of reverse causality.

The Mendelian Randomization (MR) approach is recognized as a robust method for establishing causal relationships, especially considering the growing availability of genome-wide association study results. MR leverages genetic variants, primarily single nucleotide polymorphisms (SNPs), as instrumental variables (IVs) to detect causal connections [18].

Since *N*-acetylaspartate plays multiple roles in brain homeostasis (including regulation of brain fluid balance and acts as a precursor for the neuronal dipeptide *N*-Acetylaspartylglutamate) [19,20,21,22], the aim of this study is to investigate the possible causative relationship between Cathepsin B and PD, analyzing the role of *N*-acetylaspartate as a mediator. Our study was planned after careful investigation of the previously published findings that have already linked Cathepsin B and PD. Starting from that point, however, we have tried to expand the current knowledge of this topic. The novelty of our research is the fact that we have introduced a new perspective by exploring the mediating role of *N*-acetylaspartate in association with Cathepsin B and PD. This angle has not been previously deeply investigated and represents a new step in the understanding of PD pathogenesis. The methodology that we have used for the purpose of this research is robust and might also be considered as a new way to better dissect the relationship between two phenomena. Within this context, the two-sample Mendelian randomization approach may provide new insights into the determination of possible new therapeutic strategies. For the purpose of our analysis, we have used a public and big dataset from prestigious and well-known sources. As we have already stated in the manuscript, we used summary-level data from GWAS for a two-sample Mendelian randomization analysis, exploring the association between Cathepsin B (3301 cases) and PD (4681 cases). A sequential two-step MR approach was applied (8148 cases) to study the role of *N*-acetylaspartate. This large sample size, combined with our methodological approach, minimizes the potential for bias and strengthens the reliability of our findings.

## 2. Materials and Methods

### 2.1. Study Design

**Figure 1:**
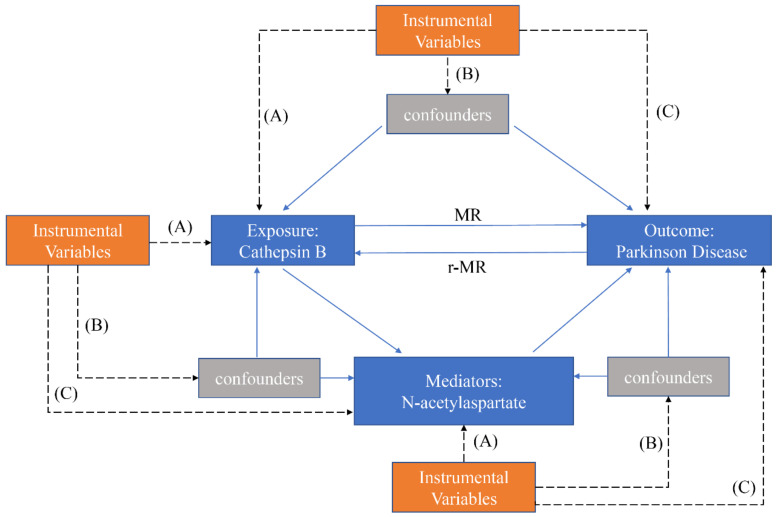
Flowchart presenting a conceptual framework for our MR analysis. (A) the IVs must exhibit a substantial association with the exposure variable; (B) the IVs should not be linked to any recognized confounding factors that might influence the relationship between the exposure and the outcome; (C) the IVs should be independent of the outcomes, exerting influence on the outcomes solely through their impact on the exposure.

The analysis performed in this study was performed using publicly accessible data, previously approved by the institutional review boards of the correspondent original studies, so there was no need for additional ethical approvals. We performed a two-sample MR framework to assess the bidirectional causality between Cathepsin B and PD. A two-step MR analysis, using SNPs as IVs, identified potential mediators, focusing particularly on *N*-acetylaspartate levels. The MR process is depicted in Figure 1.

### 2.2. Data Sources for GWAS Summary Data

The GWAS data for this analysis were obtained from publicly available databases, primarily comprising European descent cohorts (refer to Supplementary Table S1 for details). Cathepsin B genetic associations were extracted from a GWAS meta-analysis led by Sun et al. [23], encompassing 3301 European individuals (Supplementary Table S2). Parkinson’s Disease data were obtained from the FinnGen R10 study [24], which included 4681 cases and 407,500 controls (Supplementary Table S3). Data on *N*-acetylaspartate levels were retrieved from GWAS summary data by Chen et al. [25], involving 8148 individuals (Supplementary Table S4). Notably, the GWAS datasets were acquired from distinct populations, ensuring no overlap among data.

### 2.3. Instrumental Variable Selection and Data Harmonization

SNPs with genome-wide significance (*p* < 5 × 10^−5^) were selected as IVs. These SNPs were clustered considering linkage disequilibrium, applying a 10,000 kb window and an r2 value threshold below 0.001. F-statistics for each SNP calculated using the formula F = R2 (N − K − 1)/(K (1 − R2)) determined the instrumental variable strength, where R2 is the explained genetic variance in exposure, K is the number of SNPs, and N is the sample size. SNPs with an F-statistic above 10 were chosen to mitigate weak instrument bias in MR analyses.

### 2.4. Mediation Analysis

The MR analysis was conducted using R (version R-4.3.0) and the ‘Two Sample MR’ package (version 0.5.6). Outcomes were reported as odds ratios (ORs) with 95% confidence intervals (CIs) per standard deviation (SD). Mediation proportions were calculated using (β1 × β2)/β, where β represents the total effect from the primary analysis, β1 indicates Cathepsin B’s effect on the mediator, and β2 reflects the mediator’s impact on Parkinson’s Disease. Standard errors and CIs were computed using the delta method.

### 2.5. Statistical Analysis

The robustness of the causal inferences was assessed through various sensitivity analyses. These included Cochran’s Q statistics for heterogeneity statistics in the Inverse Variance Weighting (IVW) model, MR-Egger intercept tests for horizontal pleiotropy, and leave-one-out sensitivity analyses. Cochran’s Q statistics assessed heterogeneity in IVW models [26,27], indicating potential heterogeneity with significant *p* values (*p* < 0.05). The MR-Egger intercept test was applied to evaluate horizontal pleiotropy [28,29]. A non-zero intercept in the MR effect estimates of IVs implies the presence of horizontal pleiotropy. Leave-one-out analysis, recalculating the IVW estimate by sequentially excluding each SNP, was implemented to ascertain the influence of individual SNPs on the overall causal estimations [30].

## 3. Results

### 3.1. Primary Analysis

We performed various methods to assess the causal relationship between Cathepsin B and PD. The IVW method applied meta-analytical techniques to aggregate the Wald ratios representing the causal effects associated with each single SNP [31]. Complementary to IVW, methods such as MR-Egger [32] and the weighted median approach [29,33], along with simple mode and weighted mode techniques, were also utilized to increase the accuracy of the analysis.

### 3.2. Mediation Analysis

The two-step MR design for mediation analysis aimed to ascertain if *N*-acetylaspartate levels acted as a mediator in the causal pathway linking Cathepsin B to PD outcomes. This analysis dissected the overall effect into two components: the indirect effects (mediation by *N*-acetylaspartate) and the direct effects (independent of mediation). Specifically, we distinguished the total influence of Cathepsin B on PD into these direct and indirect effects, with the latter being mediated by *N*-acetylaspartate levels. To quantify the mediation, we calculated the percentage mediated by dividing the indirect effect by the total effect and provided 95% confidence intervals for this estimate, calculated using the delta method.

### 3.3. Association of Cathepsin B and Parkinson’s Disease

**Figure 2:**
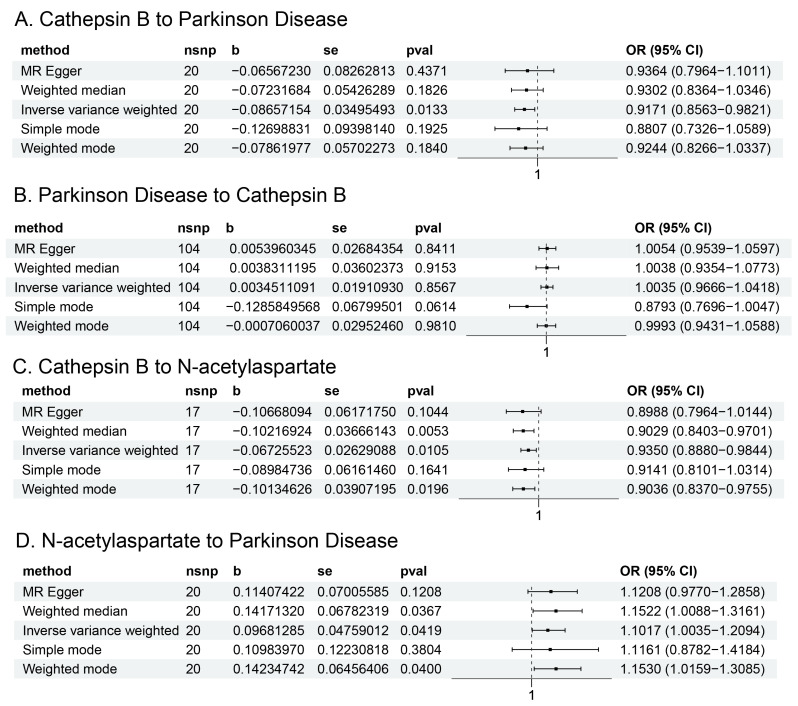
Forest plot of MR results. (**A**) The five ways used to calculate the causal relationship between Cathepsin B and Parkinson’s Disease when Cathepsin B is the exposure and Parkinson’s Disease is the outcome. MR-Egger: OR = 0.9364, 95% CI = 0.7964–1.1011, *p* = 0.4371; Weighted median: OR = 0.9302, 95% CI = 0.8364–1.0346, *p* = 0.1826; Inverse variance weighted: OR = 0.9171, 95% CI = 0.8563–0.9821, *p* = 0.0133 *; Simple mode: OR = 0.8807, 95% CI = 0.7326–1.0589, *p* = 0.1925; Weighted mode: OR = 0.9244, 95% CI = 0.8266–1.0337, *p* = 0.1840. (**B**) The five ways used to calculate the causal relationship between Parkinson’s Disease and Cathepsin B when Parkinson’s Disease is the exposure and Cathepsin B is the outcome. MR-Egger: OR = 1.0054, 95% CI = 0.9539–1.0597, *p* = 0.8411; Weighted median: OR = 1.0038, 95% CI = 0.9354–1.0773, *p* = 0.9153; Inverse variance weighted: OR = 1.0035, 95% CI = 0.9666–1.0418, *p* = 0.8567; Simple mode: OR = 0.8793, 95% CI = 0.7696–1.0047, *p* = 0.0614; Weighted mode: OR = 0.9993, 95% CI = 0.9431–1.0588, *p* = 0.9810. (**C**) The five ways used to calculate the causal relationship between Cathepsin B and *N*-acetylaspartate levels when Cathepsin B is the exposure and *N*-acetylaspartate levels are the outcome. MR-Egger: OR = 0.8988, 95% CI = 0.7964–1.0144, *p* = 0.1044; Weighted median: OR = 0.9029, 95% CI = 0.8403–0.9701, *p* = 0.0053 *; Inverse variance weighted: OR = 0.9350, 95% CI = 0.8880–0.9844, *p* = 0.0105 *; Simple mode: OR = 0.9141, 95% CI = 0.8101–1.0314, *p* = 0.1641; Weighted mode: OR = 0.9036, 95% CI = 0.8370–0.9755, *p* = 0.0196. (**D**) The five ways used to calculate the causal relationship between *N*-acetylaspartate levels and Parkinson’s Disease when *N*-acetylaspartate levels are the exposure and Parkinson’s Disease is the outcome. MR-Egger: OR = 1.1208, 95% CI = 0.9770–1.2858, *p* = 0.0122; Weighted median: OR = 1.1522, 95% CI = 1.0088–1.3161, *p* = 0.0367 *; Inverse variance weighted: OR = 1.1017, 95% CI = 1.0035–1.2094, *p* = 0.0419 *; Simple mode: OR = 1.1161, 95% CI = 0.8782–1.4184, *p* = 0.3804; Weighted mode: OR = 1.1530, 95% CI = 1.0159–1.3085, *p* = 0.0400 *. *: *p* < 0.05.

The Univariate MR analysis results concerning the relationship between Cathepsin B and Parkinson’s disease are depicted in Figure 2A, and detailed information is shown in Supplementary Table S5. A total of 20 SNPs were meticulously selected as IVs. Performing the IVW method, our analysis revealed a causal influence of Cathepsin B on PD (Odds Ratio [OR] = 0.9171, 95% Confidence Interval [CI] = 0.8563–0.9821, *p* = 0.0133). This suggested that higher levels of Cathepsin B are associated with lower odds (about 8.3% reduction) of developing PD. Symmetric funnel plots lent credence to the absence of significant bias in SNP selection, as shown in Supplementary Figure S1A. The scatter plot depicts the causal relationship between Cathepsin B and PD by the line’s slope, which varies depending on the MR tests, as shown in Supplementary Figure S1B. The causal association between Cathepsin B and PD is assessed by IVW approaches for each individual SNP, as shown in Supplementary Figure S1C. The forest plot depicts the leave-one-out analysis. Every dot signifies the MR estimate result using IVW that does not include that specific SNP, as shown in Supplementary Figure S1D. The characteristics of the Cathepsin B-related genetic variants and their effects on PD (20 SNPs) are shown in Supplementary Table S7. Further, we discovered that CTSB was downregulated in PD, as seen in Supplementary Figure S2.

The Univariate MR analysis concerning the influence of PD on Cathepsin B was presented in Figure 2B and Supplementary Table S5. In this case, a total of 104 SNPs served as IVs. Intriguingly, even employing the IVW method, the resultant *p*-value exceeded the conventional significance threshold of 0.05 (*p* = 0.8567). The same result was obtained by MR-Egger, Weighted median, Simple mode, and Weighted mode methods. The leave-one-out analyses plot, funnel plot, scatter plot, and forest plot of individual SNPs showing the association between PD and Cathepsin B was presented in Supplementary Figure S3.

### 3.4. Association of Cathepsin B with N-Acetylaspartate Levels

We identified a total of 17 genome-wide significant SNPs to serve as IVs for the purpose of this analysis. Performing the IVW, Weighted median, and Weighted mode methods, we observed a positive association between genetically predicted Cathepsin B and the risk of *N*-acetylaspartate levels (IVW method: OR = 0.9350, 95% CI = 0.8880–0.9844, *p* = 0.0105; Weighted median method: OR = 0.9029, 95% CI = 0.8403–0.9701, *p* = 0.0053; Weighted mode method: OR = 0.9036, 95% CI = 0.8370–0.9755, *p* = 0.0196). This suggested that higher levels of Cathepsin B are associated with a decrease (about 6.5%) in *N*-acetylaspartate levels. The results were reported in Figure 2C and are shown in Supplementary Table S5. Symmetric funnel plots lent credence to the absence of significant bias in SNP selection, as shown in Supplementary Figure S3A. The scatter plot depicted the causal relationship between Cathepsin B and *N*-acetylaspartate level by the line’s slope, which varies depending on the MR tests, as shown in Supplementary Figure S3B. The causal association between Cathepsin B and *N*-acetylaspartate level is assessed by IVW approaches for each individual SNP, as shown in Supplementary Figure S3C. The forest plot depicts the leave-one-out analysis. Every dot signifies MR estimate results using IVW that do not include that specific SNP, as shown in Supplementary Figure S3D. Characteristics of the Cathepsin B-related genetic variants and their effects on *N*-acetylaspartate (17 SNPs) are shown in Supplementary Table S8.

### 3.5. Association of N-Acetylaspartate Levels with PD

In Supplementary Table S4, we have comprehensively presented all genetic instruments linked to *N*-acetylaspartate levels. As represented in Figure 2D and Supplementary Table S5, genetically predicted *N*-acetylaspartate levels exhibited a significant positive correlation with PD (IVW method: OR = 1.1017, 95% CI: 1.0035–1.2094; *p* = 0.0419; Weighted median method: OR = 1.1522, 95% CI: 1.0088–1.3161; *p* = 0.0367; Weighted mode method: OR = 1.1530, 95% CI: 1.0159−1.3085; *p* = 0.0400). The leave-one-out analysis plot, funnel plot, scatter plot, and forest plot of individual SNPs showing the association between *N*-acetylaspartate levels and PD are presented in Supplementary Figure S3.

### 3.6. Proportion of the Association between Cathepsin B and PD Mediated by N-Acetylaspartate Levels

Our study investigated the potential mediating role of *N*-acetylaspartate levels in the pathway from Cathepsin B to PD. Our analysis detected an association wherein Cathepsin B was linked to reduced *N*-acetylaspartate levels, subsequently contributing to an elevated risk of PD. Our study elucidated that *N*-acetylaspartate levels accounted for 9.87% of the heightened risk of PD attributed to Cathepsin B (proportion mediated: 7.52%, 95% CI = −3.65% to 18.69%).

### 3.7. Sensitivity Analysis

The Cochran’s Q tests conducted in our study indicated an absence of significant heterogeneity among the IVs utilized for MR analysis. The MR-Egger regression intercept analysis revealed that all *p*-values were greater than 0.05, suggesting no substantial horizontal pleiotropy. During the MR-PRESSO testing phase, outliers with notable levels of pleiotropy were identified and excluded. Subsequently, the MR analysis was re-executed with the refined set of SNPs. The reliability of our primary findings was further reinforced through a leave-one-out sensitivity analysis. Detailed information pertaining to these results is provided in Supplementary Table S6.

## 4. Discussion

Our research investigated the intricate relationship between Cathepsin B and PD, with a specific focus on examining the mediating role of *N*-acetylaspartate. The use of two-sample MR and the two-step MR approach, incorporating data from existing European cohort studies, provided a robust methodology that may help to better understand this putative correlation.

Previous studies showed that Cathepsin B, a lysosomal hydrolase, could be investigated as a potential biomarker and risk factor for the development of PD [11]. The association between Cathepsin B and PD has been implied by genetic [12,13], clinical [30], and experimental studies [31,32]. Indeed, researches across various populations have confirmed the possible genetic link between Cathepsin B and PD. Chang and colleagues conducted a GWAS involving 6476 participants from the 23 and Me PD cohort (PDWBS—Web-Based Study of Parkinson’s Disease) and 302,042 control individuals genotyped using custom Illumina arrays. This study identified that common variants in the CTSB gene, which encodes Cathepsin B, were associated with an increased risk of developing PD [15]. Similarly, Milanowski and collaborators performed a WES analysis on individuals from a family affected by PD, discovering that a rare variant (p.Gly284Val) in the CTSB gene might be responsible for the PD symptoms [16]. The authors described the genetic basis of PD in a Polish family with two affected siblings, and they conducted a preliminary functional analysis of the identified CTSB mutation in fibroblasts derived from the patients, expanding the knowledge of gene expression data for brain tissues [16]. Furthermore, Jones-Tabah and colleagues analyzed genetic data from multiple sources, including the Fox Investigation for New Discovery of Biomarkers (BioFIND), the Harvard Biomarker Study (HBS), the Parkinson’s Progression Markers Initiative (PPMI), the Parkinson’s Disease Biomarkers Program (PDBP), the International LBD Genomics Consortium (ILBDGC), and the STEADY-PD III Investigators. Their findings were in line with the other studies since they also suggested that rare variations in the CTSB gene might contribute to an increased risk of PD [17]. Sjödin and colleagues conducted a pilot study combining solid-phase extraction and parallel reaction monitoring mass spectrometry and discovered that the concentration of Cathepsin B decreased [34]. The work by McGlinchey and collaborators implicated Cathepsin B as essential in α-synuclein lysosomal degradation [35,36]. Remarkably, Jones-Tabah and colleagues performed various experiments to demonstrate that Cathepsin B might enhance the clearance of fibrillar alpha-synuclein, increase lysosomal functionality, and boost glucocerebrosidase activity in dopaminergic neurons [17].

The results from our MR analysis intriguingly suggested a protective causal relationship between elevated levels of genetically predicted Cathepsin B and a decreased likelihood of developing PD. This finding is particularly noteworthy in light of the conventional understanding of PD pathophysiology. Indeed, our study extended this paradigm by highlighting the potential role of lysosomal dysfunction. A robust association between PD and lysosomal dysfunction has already been detected as a crucial point in the pathogenesis models of PD [37]. Indeed, lysosomal storage disorders, characterized by the impairment of lysosomal proteins, share pathological features with PD [38]. In this context, it is noteworthy that the participation of lysosomal enzymes, including glucocerebrosidase (encoded by *GBA1*), can significantly influence the pathogenesis of PD [39,40,41,42,43]. Within the 90 identified loci associated with PD susceptibility [44], variants in the *GBA1* gene, recognized as the most common genetic risk factor in PD, have gained significant interest in the field [45,46]. Data indicated that approximately 5% of individuals diagnosed with PD had a mutation in the *GBA1* gene [47]. The presence of either heterozygous or homozygous mutations in *GBA1* was correlated with a 20- to 30-fold increase in the risk of PD onset [48,49]. Deficient glucocerebrosidase function impaired lysosomal activity, resulting in the reduced catabolism of α-synuclein, facilitating its aggregation and the consequent neurodegenerative cascade [50].

Moreover, mediation analysis revealed the role of *N*-acetylaspartate in the association between Cathepsin B and PD, albeit as a minor player. *N*-acetylaspartate has been involved in several cellular processes, including osmoregulation in neurons, supplying acetate for myelin lipid synthesis, production of the neuropeptide *N*-acetylaspartylglutamate, and supporting energy metabolism in neuronal mitochondria [21]. Recent studies have suggested that *N*-acetylaspartate may have a potential role in stabilizing proteins and inhibiting protein aggregation [51]. Within this context, Gröger and colleagues found significantly decreased *N*-acetylaspartate concentrations in the substantia nigra of PD patients compared to controls, using three-dimensional magnetic resonance spectroscopic imaging [52]. Further studies are needed to better evaluate these findings in order to understand the intricate pathophysiology of PD.

The current study has limitations. We focused on European individuals, so data from other populations of patients are needed to confirm the present results. This would be possible once more GWAS data including information about Cathepsin B and *N*-acetylaspartate from non-European individuals are available. Furthermore, the specific biological functions of many SNPs remain to be fully understood. Despite these limitations, our research sheds more light on the relationship between Cathepsin B and PD, providing a new base for future investigative directions. The MR methodology is notably less susceptible to confounding factors and reverse causation compared to traditional observational and interventional studies. However, further investigation and data are required to better elucidate the causal correlation between Cathepsin B and PD. Another limitation of our study is that we have focused only on one type of Cathepsin, so further studies should investigate the possible association between PD and other kinds of Cathepsins, such as Cathepsin D and Cathepsin L [14].

## 5. Conclusions

Our study increased the current knowledge in the understanding of PD pathogenesis. The study confirmed the possible role of Cathepsin B as a biomarker and risk factor for PD development and opened new perspectives on the metabolic pathways involved in the disease. Although it seemed to have a limited role, the mediating effect of *N*-acetylaspartate provided a new hint for further studies.

## Data Availability

The GWAS summary statistics were deposited to the GWAS Catalog (https://www.ebi.ac.uk/gwas/, accessed on 10 January 2024).

## Supplementary Materials

The following supporting information can be downloaded at: https://www.mdpi.com/article/10.3390/brainsci14050482/s1, Supplementary Figures: Supplementary Figure S1: Relationship examination between Cathepsin B and PD. Supplementary Figure S2: Relationship examination between Cathepsin B and *N*-acetylaspartate level. Supplementary Figure S3: Relationship examination between *N*-acetylaspartate level and PD. Supplementary Tables: Supplementary Table S1: Detailed information for databases regarding Cathepsin B, Parkinson’s Disease, *N*-acetylaspartate (naa) levels. Supplementary Table S2: Instrumental variables used in MR analysis regarding Cathepsin B (*p* < 5 × 10^−5^). Supplementary Table S3: Instrumental variables used in MR analysis regarding Parkinson’s Disease (*p* < 5 × 10^−5^). Supplementary Table S4: Instrumental variables used in MR analysis regarding *N*-acetylaspartate (naa) levels (*p* < 5 × 10^−5^). Supplementary Table S5: Effect estimates of the association between Cathepsin B, *N*-acetylaspartate (naa) levels, and Parkinson’s Disease after excluding outliers SNPs. Supplementary Table S6: Evaluation of heterogeneity and horizontal pleiotropy using different methods. Supplementary Table S7: Characteristic of the Cathepsin B-related genetic variants and their effects on Parkinson’s Disease (20 SNPs). Supplementary Table S8: Characteristic of the Cathepsin B-related genetic variants and their effects on *N*-acetylaspartate (17 SNPs). Supplementary Table S9: Characteristic of the *N*-acetylaspartate-related genetic variants and their effects on Parkinson’s Disease (20 SNPs).

## References

[1] Poewe W., Seppi K., Tanner C.M., Halliday G.M., Brundin P., Volkmann J., Schrag A.-E., Lang A.E. (2017). Parkinson disease. Nat. Rev. Dis. Primers.

[2] Wirdefeldt K., Adami H.O., Cole P., Trichopoulos D., Mandel J. (2011). Epidemiology and etiology of Parkinson’s disease: A review of the evidence. Eur. J. Epidemiol..

[3] Tysnes O.B., Storstein A. (2017). Epidemiology of Parkinson’s disease. J. Neural Transm..

[4] Shulman L.M. (2010). Understanding disability in Parkinson’s disease. Mov. Disord. Off. J. Mov. Disord. Soc..

[5] Geut H., Hepp D.H., Foncke E., Berendse H.W., Rozemuller J.M., Huitinga I., van de Berg W.D.J. (2020). Neuropathological correlates of parkinsonian disorders in a large Dutch autopsy series. Acta Neuropathol. Commun..

[6] Belvisi D., Pellicciari R., Fabbrini A., Costanzo M., Ressa G., Pietracupa S., De Lucia M., Modugno N., Magrinelli F., Dallocchio C. (2022). Relationship between risk and protective factors and clinical features of Parkinson’s disease. Park. Relat. Disord..

[7] Ercoli T., Masala C., Cadeddu G., Mascia M.M., Orofino G., Gigante A.F., Solla P., Defazio G., Rocchi L. (2022). Does Olfactory Dysfunction Correlate with Disease Progression in Parkinson’s Disease? A Systematic Review of the Current Literature. Brain Sci..

[8] Masala C., Solla P., Liscia A., Defazio G., Saba L., Cannas A., Cavazzana A., Hummel T., Haehner A. (2018). Correlation among olfactory function, motors’ symptoms, cognitive impairment, apathy, and fatigue in patients with Parkinson’s disease. J. Neurol..

[9] Váradi C. (2020). Clinical Features of Parkinson’s Disease: The Evolution of Critical Symptoms. Biology.

[10] DeMaagd G., Philip A. (2015). Parkinson’s Disease and Its Management: Part 1: Disease Entity, Risk Factors, Pathophysiology, Clinical Presentation, and Diagnosis. Pharm. Ther..

[11] Goldenberg M.M. (2008). Medical management of Parkinson’s disease. Pharm. Ther..

[12] Navarro-Romero A., Montpeyó M., Martinez-Vicente M. (2020). The Emerging Role of the Lysosome in Parkinson’s Disease. Cells.

[13] Drobny A., Boros F.A., Balta D., Prieto Huarcaya S., Caylioglu D., Qazi N., Vandrey J., Schneider Y., Dobert J.P., Pitcairn C. (2023). Reciprocal effects of alpha-synuclein aggregation and lysosomal homeostasis in synucleinopathy models. Transl. Neurodegener..

[14] Drobny A., Prieto Huarcaya S., Dobert J., Kluge A., Bunk J., Schlothauer T., Zunke F. (2022). The role of lysosomal cathepsins in neurodegeneration: Mechanistic insights, diagnostic potential and therapeutic approaches. Biochim. Biophys. Acta (BBA) Mol. Cell Res..

[15] Chang D., Nalls M.A., Hallgrímsdóttir I.B., Hunkapiller J., van der Brug M., Cai F., Kerchner G.A., Ayalon G., Bingol B., Sheng M. (2017). A meta-analysis of genome-wide association studies identifies 17 new Parkinson’s disease risk loci. Nat. Genet..

[16] Milanowski L.M., Hou X., Bredenberg J.M., Fiesel F.C., Cocker L.T., Soto-Beasley A.I., Walton R.L., Strongosky A.J., Faroqi A.H., Barcikowska M. (2022). Cathepsin B p.Gly284Val Variant in Parkinson’s Disease Pathogenesis. Int. J. Mol. Sci..

[17] Jones-Tabah J., He K., Senkevich K., Karpilovsky N., Deyab G., Cousineau Y., Nikanorova D., Goldsmith T., Del Cid Pellitero E., Chen C.X. (2023). The Parkinson’s disease risk gene cathepsin B promotes fibrillar alpha-synuclein clearance, lysosomal function and glucocerebrosidase activity in dopaminergic neurons. bioRxiv.

[18] Sanderson E., Glymour M.M., Holmes M.V., Kang H., Morrison J., Munafò M.R., Palmer T., Schooling C.M., Wallace C., Zhao Q. (2022). Mendelian randomization. Nat. Rev. Methods Primers.

[19] Griffith H.R., den Hollander J.A., Okonkwo O.C., O’Brien T., Watts R.L., Marson D.C. (2008). Brain *N*-acetylaspartate is reduced in Parkinson disease with dementia. Alzheimer Dis. Assoc. Disord..

[20] Pan J.W., Takahashi K. (2005). Interdependence of *N*-acetyl aspartate and high-energy phosphates in healthy human brain. Ann. Neurol..

[21] Moffett J.R., Ross B., Arun P., Madhavarao C.N., Namboodiri A.M. (2007). *N*-Acetylaspartate in the CNS: From neurodiagnostics to neurobiology. Prog. Neurobiol..

[22] Arun P., Madhavarao C.N., Moffett J.R., Namboodiri M.A. (2006). Regulation of *N*-acetylaspartate and *N*-acetylaspartylglutamate biosynthesis by protein kinase activators. J. Neurochem..

[23] Sun B.B., Maranville J.C., Peters J.E., Stacey D., Staley J.R., Blackshaw J., Burgess S., Jiang T., Paige E., Surendran P. (2018). Genomic atlas of the human plasma proteome. Nature.

[24] Kurki M.I., Karjalainen J., Palta P., Sipilä T.P., Kristiansson K., Donner K.M., Reeve M.P., Laivuori H., Aavikko M., Kaunisto M.A. (2023). FinnGen provides genetic insights from a well-phenotyped isolated popu lation. Nature.

[25] Chen Y., Lu T., Pettersson-Kymmer U., Stewart I.D., Butler-Laporte G., Nakanishi T., Cerani A., Liang K.Y.H., Yoshiji S., Willett J.D.S. (2023). Genomic atlas of the plasma metabolome prioritizes metabolites implicated in human diseases. Nat. Genet..

[26] Huedo-Medina T.B., Sánchez-Meca J., Marín-Martínez F., Botella J. (2006). Assessing heterogeneity in meta-analysis: Q statistic or I2 index?. Psychol. Methods.

[27] Greco M.F., Minelli C., Sheehan N.A., Thompson J.R. (2015). Detecting pleiotropy in Mendelian randomisation studies with summary data and a continuous outcome. Stat. Med..

[28] Verbanck M., Chen C.Y., Neale B., Do R. (2018). Detection of widespread horizontal pleiotropy in causal relationships inferred from Mendelian randomization between complex traits and diseases. Nat. Genet..

[29] Bowden J., Davey Smith G., Burgess S. (2015). Mendelian randomization with invalid instruments: Effect estimation and bias detection through Egger regression. Int. J. Epidemiol..

[30] Burgess S., Davey Smith G., Davies N.M., Dudbridge F., Gill D., Glymour M.M., Hartwig F.P., Kutalik Z., Holmes M.V., Minelli C. (2019). Guidelines for performing Mendelian randomization investigations: Update for summer 2023. Wellcome Open Res..

[31] Burgess S., Butterworth A., Thompson S.G. (2013). Mendelian randomization analysis with multiple genetic variants using summarized data. Genet. Epidemiol..

[32] Minelli C., Del Greco M.F., van der Plaat D.A., Bowden J., Sheehan N.A., Thompson J. (2021). The use of two-sample methods for Mendelian randomization analyses on single large datasets. Int. J. Epidemiol..

[33] Bowden J., Davey Smith G., Haycock P.C., Burgess S. (2016). Consistent Estimation in Mendelian Randomization with Some Invalid Instruments Using a Weighted Median Estimator. Genet. Epidemiol..

[34] Sjödin S., Brinkmalm G., Öhrfelt A., Parnetti L., Paciotti S., Hansson O., Hardy J., Blennow K., Zetterberg H., Brinkmalm A. (2019). Endo-lysosomal proteins and ubiquitin CSF concentrations in Alzheimer’s and Parkinson’s disease. Alzheimer’s Res. Ther..

[35] McGlinchey R.P., Lee J.C. (2015). Cysteine cathepsins are essential in lysosomal degradation of α-synuclein. Proc. Natl. Acad. Sci. USA.

[36] McGlinchey R.P., Lacy S.M., Huffer K.E., Tayebi N., Sidransky E., Lee J.C. (2019). C-terminal α-synuclein truncations are linked to cysteine cathepsin activity in Parkinson’s disease. J. Biol. Chem..

[37] Fraldi A., Klein A.D., Medina D.L., Settembre C. (2016). Brain Disorders Due to Lysosomal Dysfunction. Annu. Rev. Neurosci..

[38] Shachar T., Lo Bianco C., Recchia A., Wiessner C., Raas-Rothschild A., Futerman A.H. (2011). Lysosomal storage disorders and Parkinson’s disease: Gaucher disease and beyond. Mov. Disord. Off. J. Mov. Disord. Soc..

[39] Beavan M., McNeill A., Proukakis C., Hughes D.A., Mehta A., Schapira A.H. (2015). Evolution of prodromal clinical markers of Parkinson disease in a GBA mutation-positive cohort. JAMA Neurol..

[40] Do J., McKinney C., Sharma P., Sidransky E. (2019). Glucocerebrosidase and its relevance to Parkinson disease. Mol. Neurodegener..

[41] Sidransky E., Nalls M.A., Aasly J.O., Aharon-Peretz J., Annesi G., Barbosa E.R., Bar-Shira A., Berg D., Bras J., Brice A. (2009). Multicenter analysis of glucocerebrosidase mutations in Parkinson’s disease. N. Engl. J. Med..

[42] Huh Y.E., Usnich T., Scherzer C.R., Klein C., Chung S.J. (2023). GBA1 Variants and Parkinson’s Disease: Paving the Way for Targeted Therapy. J. Mov. Disord..

[43] Olszewska D.A., Lynch T. (2018). Lysosomal Storage Disorders and Parkinson’s Disease: New Susceptibility Loci Identified. Mov. Disord. Clin. Pract..

[44] Nalls M.A., Blauwendraat C., Vallerga C.L., Heilbron K., Bandres-Ciga S., Chang D., Tan M., Kia D.A., Noyce A.J., Xue A. (2019). Identification of novel risk loci, causal insights, and heritable risk for Parkinson’s disease: A meta-analysis of genome-wide association studies. Lancet Neurol..

[45] Saunders-Pullman R., Hagenah J., Dhawan V., Stanley K., Pastores G., Sathe S., Tagliati M., Condefer K., Palmese C., Brüggemann N. (2010). Gaucher disease ascertained through a Parkinson’s center: Imaging and clinical characterization. Mov. Disord. Off. J. Mov. Disord. Soc..

[46] Aharon-Peretz J., Badarny S., Rosenbaum H., Gershoni-Baruch R. (2005). Mutations in the glucocerebrosidase gene and Parkinson disease: Phenotype-genotype correlation. Neurology.

[47] Alcalay R.N., Levy O.A., Waters C.C., Fahn S., Ford B., Kuo S.H., Mazzoni P., Pauciulo M.W., Nichols W.C., Gan-Or Z. (2015). Glucocerebrosidase activity in Parkinson’s disease with and without GBA mutations. Brain.

[48] Steger M., Tonelli F., Ito G., Davies P., Trost M., Vetter M., Wachter S., Lorentzen E., Duddy G., Wilson S. (2016). Phosphoproteomics reveals that Parkinson’s disease kinase LRRK2 regulates a subset of Rab GTPases. eLife.

[49] Ito G., Katsemonova K., Tonelli F., Lis P., Baptista M.A., Shpiro N., Duddy G., Wilson S., Ho P.W., Ho S.L. (2016). Phos-tag analysis of Rab10 phosphorylation by LRRK2: A powerful assay for assessing kinase function and inhibitors. Biochem. J..

[50] Smith L.J., Lee C.Y., Menozzi E., Schapira A.H.V. (2022). Genetic variations in GBA1 and LRRK2 genes: Biochemical and clinical consequences in Parkinson disease. Front. Neurol..

[51] Warepam M., Mishra A.K., Sharma G.S., Kumari K., Krishna S., Khan M.S.A., Rahman H., Singh L.R. (2021). Brain Metabolite, *N*-Acetylaspartate Is a Potent Protein Aggregation Inhibitor. Front. Cell. Neurosci..

[52] Gröger A., Kolb R., Schäfer R., Klose U. (2014). Dopamine Reduction in the Substantia Nigra of Parkinson’s Disease Patients Confirmed by In Vivo Magnetic Resonance Spectroscopic Imaging. PLoS ONE.

