# Supplementary material for: Exploring the Association between Cathepsin B and Parkinson’s Disease
Source: Brain Sci. 2024 May 10;14(5):482. doi: 10.3390/brainsci14050482 (PMC11119263; doi:10.3390/brainsci14050482)
Supplement: Supplementary file 1 [file brainsci-14-00482-s001.zip › Supplementary Figures.pdf]

Supplementary Figures

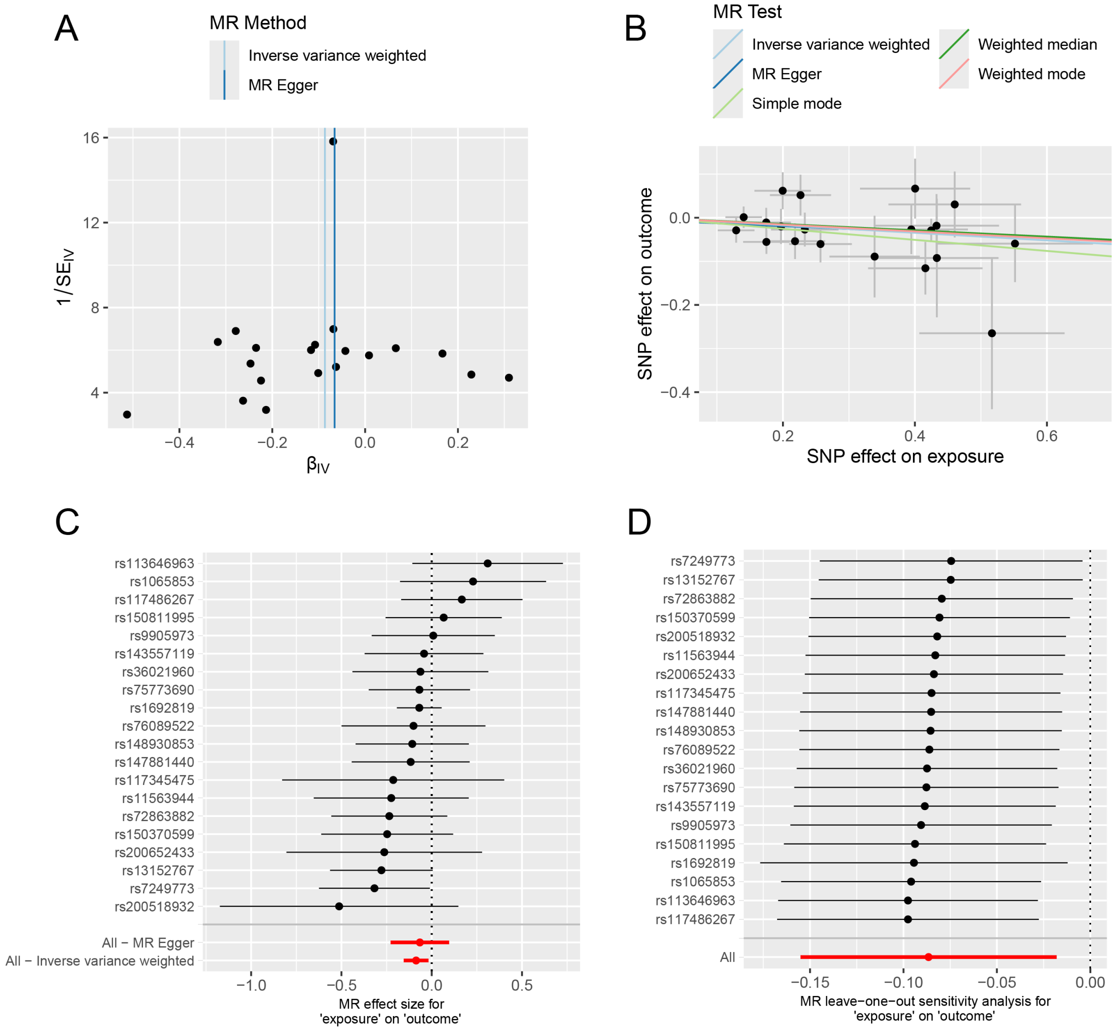

Supplementary Figure S1. Relationship examination between Cathepsin B and PD.

(A) Funnel plot was used to illustrate the individual variation effects for the instrument variables shown against the inverse of their standard error.

(B) Scatter plot depicted the causal relationship between Cathepsin B and PD by the line's slope, which varies depending on the MR tests.

(C) Forest plot was used to illustrate how Cathepsin B is associated with a decreased risk of PD. The causal association between Cathepsin B and PD is assessed by IVW approaches for each individual SNP.

(D)Forest plot depicted leave-one-out analysis. Every dot signified the MR estimate result using IVW that does not include that specific SNP.

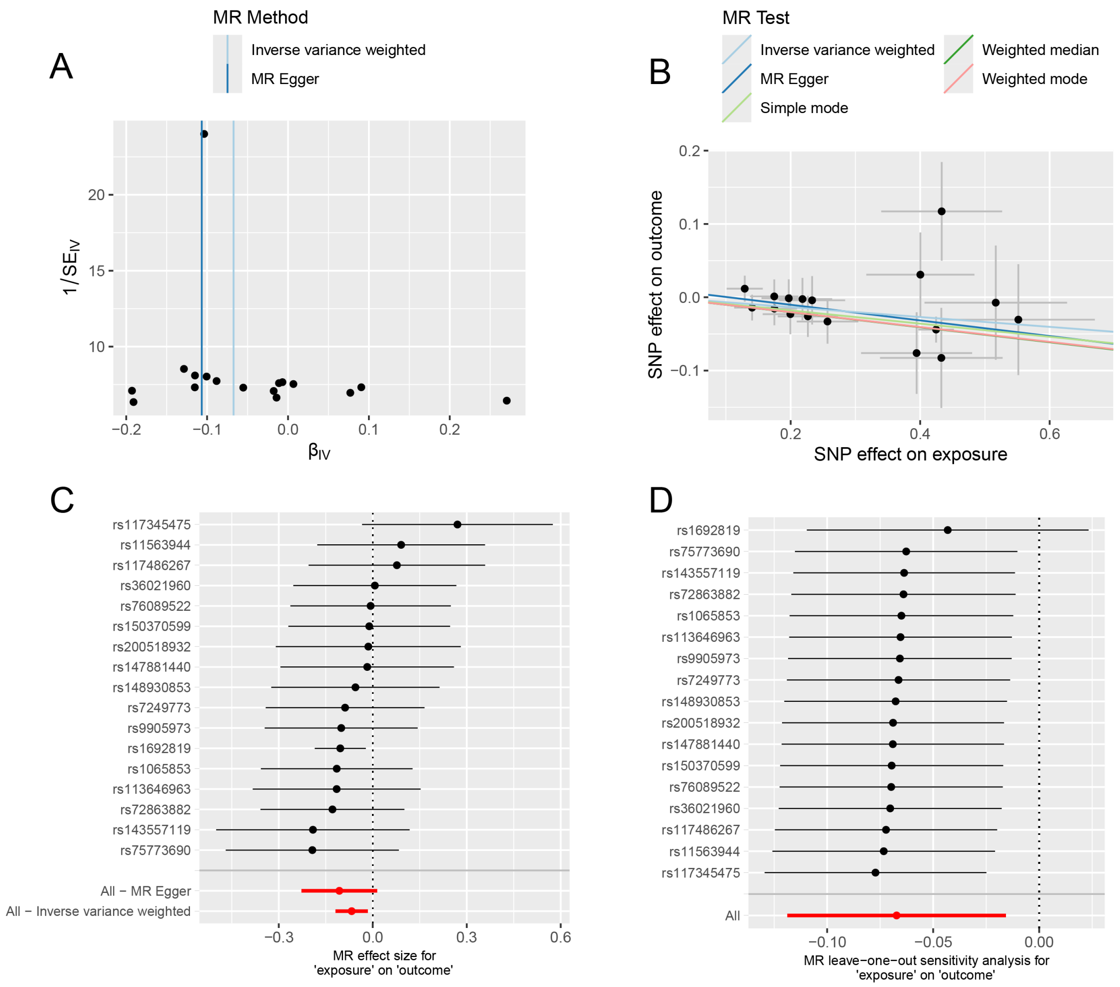

Supplementary Figure S2. Relationship examination between Cathepsin B and N-acetylaspartate level.

(A)Funnel plot was used to illustrate the individual variation effects for the instrument variables shown against the inverse of their standard error.

(B)Scatter plot depicted the causal relationship between Cathepsin B and N-acetylaspartate level by the line's slope, which varies depending on the MR tests.

(C)Forest plot was used to illustrate how Cathepsin B is associated with a decreased risk of N-acetylaspartate level. The causal association between Cathepsin B and N-acetylaspartate level is assessed by IVW approaches for each individual SNP.

(D)Forest plot depicted leave-one-out analysis. Every dot signified the MR estimate result using IVW that does not include that specific SNP.

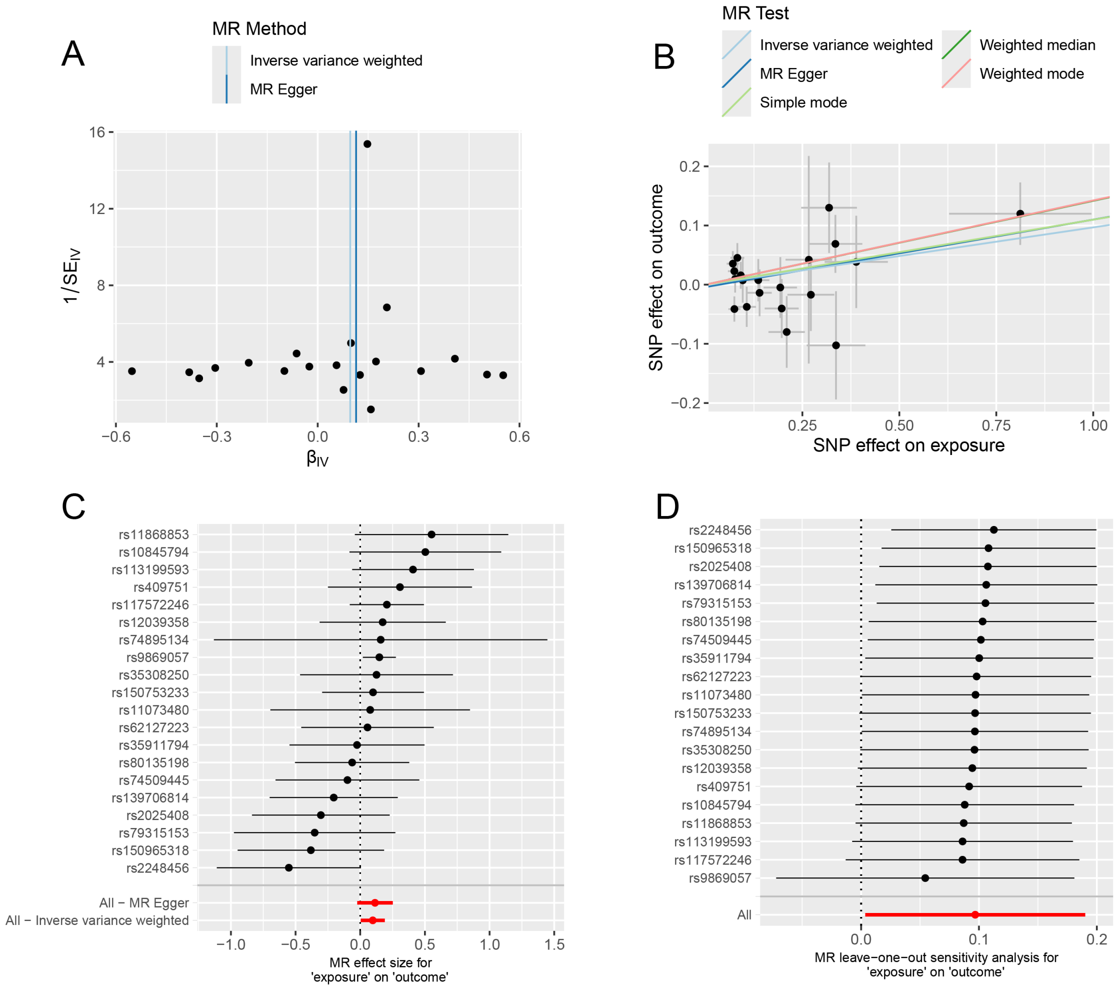

Supplementary Figure S3. Relationship examination between N-acetylaspartate level and PD.

(A) Funnel plot was used to illustrate the individual variation effects for the instrument variables shown against the inverse of their standard error.

(B) Scatter plot depicted the causal relationship between N-acetylaspartate level and PD by the line's slope, which varies depending on the MR tests.

(C) Forest plot was used to illustrate how N-acetylaspartate level is associated with an increased risk of PD. The causal association between N-acetylaspartate level and PD is assessed by IVW approaches for each individual SNP.

(D) Forest plot depicted leave-one-out analysis. Every dot signified the MR estimate result using IVW that does not include that specific SNP.
